# Treatment with FRAX486 rescues neurobehavioral and metabolic alterations in a female mouse model of CDKL5 deficiency disorder

**DOI:** 10.1111/cns.13907

**Published:** 2022-08-06

**Authors:** Claudia Fuchs, Livia Cosentino, Chiara Urbinati, Maria Cristina Talamo, Giorgio Medici, Maria Cristina Quattrini, Nicola Mottolese, Donatella Pietraforte, Andrea Fuso, Elisabetta Ciani, Bianca De Filippis

**Affiliations:** ^1^ Department of Biomedical and Neuromotor Science University of Bologna Bologna Italy; ^2^ Center for Behavioral Sciences and Mental Health Istituto Superiore di Sanità Rome Italy; ^3^ Core Facilities, Istituto Superiore di Sanità Rome Italy; ^4^ Department of Experimental Medicine Sapienza University of Rome Rome Italy

**Keywords:** animal model, behavior, CDKL5 deficiency disorder, cytoskeleton, therapeutic approach, transfection

## Abstract

**Introduction:**

CDKL5 deficiency disorder (CDD) is a rare neurodevelopmental condition, primarily affecting girls for which no cure currently exists. Neuronal morphogenesis and plasticity impairments as well as metabolic dysfunctions occur in CDD patients. The present study explored the potential therapeutic value for CDD of FRAX486, a brain‐penetrant molecule that was reported to selectively inhibit group I p21‐activated kinases (PAKs), serine/threonine kinases critically involved in the regulation of neuronal morphology and glucose homeostasis.

**Methods:**

The effects of treatment with FRAX486 on CDD‐related alterations were assessed in vitro (100 nM for 48 h) on primary hippocampal cultures from *Cdkl5*‐knockout male mice (*Cdkl5*‐KO) and in vivo (20 mg/Kg, s.c. for 5 days) on *Cdkl5*‐KO heterozygous females (*Cdkl5*‐Het).

**Results:**

The in vitro treatment with FRAX486 completely rescued the abnormal neuronal maturation and the number of PSD95‐positive puncta in *Cdkl5*‐KO mouse neurons. In vivo, FRAX486 normalized the general health status, the hyperactive profile and the fear learning defects of fully symptomatic *Cdkl5*‐Het mice. Systemically, FRAX486 treatment normalized the levels of reactive oxidizing species in the whole blood and the fasting‐induced hypoglycemia displayed by *Cdkl5*‐Het mice. In the hippocampus of *Cdkl5*‐Het mice, treatment with FRAX486 rescued spine maturation and PSD95 expression and restored the abnormal PAKs phosphorylation at sites which are critical for their activation (P‐PAK‐Ser144/141/139) or for the control cytoskeleton remodeling (P‐PAK1‐Thr212).

**Conclusions:**

Present results provide evidence that PAKs may represent innovative therapeutic targets for CDD.

## Graphical abstract

CDKL5 deficiency disorder (CDD) is a severe neurological disease for which no cure exists. A systemic 5‐day long treatment with FRAX486 rescued both the neurobehavioral alterations and hippocampal spine imbalance in a female mouse model of CDD at a clearly symptomatic stage. In mouse blood, FRAX486 rescued the increased oxidative stress status and the fasting‐induced hypoglycemia. Importantly, the treatment also restored the normal activity of PAKs kinase family.

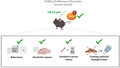

## 1 INTRODUCTION

CDKL5 deficiency disorder (CDD) (OMIM #300672) is a rare neurodevelopmental condition affecting primarily females, with a ~4:1 female‐to‐male ratio^1^ characterized by a variety of behavioral and physiological symptoms that include the onset of seizures within the first weeks/months of life, severe global developmental delay resulting in intellectual disability, poor motor control and the presence of peculiar hand stereotypies.^2^, ^3^ To date, no cure to improve/ameliorate brain development and behavioral abnormalities in CDD patients exists.

CDKL5 deficiency disorder is a X‐linked dominant disorder caused by de novo mutations in the *cyclin‐dependent kinase‐like 5* (*CDKL5*) gene and most patients are females heterozygous for the mutations.^4^ *CDKL5* encodes a serine/threonine kinase expressed in various tissues, with the brain showing the highest levels of expression. Available data point to a crucial role of CDKL5 in fundamental neurodevelopmental processes such as activity‐dependent regulation of neuronal morphogenesis and plasticity.^5^ Indeed, various microtubule and actin binding proteins have been identified as interactors of CDKL5,^6^, ^7^, ^8^ indicating that its roles may converge on regulating cytoskeleton dynamics. In this line, reduced dendritic arborization of cortical and hippocampal neurons and abnormal morphology and density of dendritic spines have been identified both in mice lacking Cdkl5 (*Cdkl5*‐KO) and in induced‐pluripotent stem cells (iPSCs)‐derived neurons from CDD patients.^8^

A role for CDKL5 in the regulation of energy metabolism is also emerging. Recent findings in fact provide evidence that mitochondrial dysfunction and oxidative stress occur in CDD.^9^, ^10^, ^11^ These include altered lipid profile in fibroblasts and systemic redox imbalance in plasma samples from CDD patients.^9^ Reduced activity of mitochondrial respiratory chain complexes in *Cdkl5*‐KO brains was also found, which was accompanied by an impairment in mitochondrial ATP production rate and reduced ATP whole brain levels.^11^ However, the underlying mechanisms and their role in the pathogenesis of CDD have not been clarified.

Since loss of CDKL5 affects neuronal morphology and a role for CDKL5 in the regulation of cytoskeleton dynamics has been highlighted, in the present study we focused our attention on group I p21‐activated kinases (PAKs), a family of serine/threonine kinases (PAK1/2/3) that are critically involved in the regulation of microtubule and actin‐cytoskeleton remodeling.^12^ In neurons, PAKs control the activity of actin‐binding proteins that stimulate or inhibit actin polymerization, branching, and stabilization, thus critically regulating the organization of F‐actin in spines.^13^, ^14^ Moreover, PAKs bind to microtubule‐stabilizing proteins, thus affecting microtubule organization and dynamics.^15^, ^16^, ^17^, ^18^ Consistently, PAKs play a crucial role in structural neuronal changes that underlie synaptic plasticity^19^ and prolonged activation of this family of proteins results in spine shrinkage in brains and neuronal cultures.^20^, ^21^ Recent evidence also demonstrates that PAKs overactivation negatively affects fear memory,^22^ social learning,^23^ and sensory processing.^19^, ^24^ In this line, inhibitors of PAKs are able to improve/rescue intellectual deficits, motor dysfunction, autistic‐like behaviors, and spine deterioration in preclinical models of Fragile X and schizophrenia.^20^, ^25^, ^26^

Interestingly, PAKs are also emerging as regulators of glucose homeostasis in peripheral tissues as well as in neurons.^27^ In particular, PAKs were shown to modulate insulin‐mediated glucose uptake through cytoskeleton remodeling, an essential mechanism for maintaining a steady balance between nutrient supply and energy demand, and preserving higher brain functions, including cognition.^28^ In fact, cognitive dysfunction in PAKs‐KO mice is accompanied by impaired glucose disposal, abnormalities in plasma levels of metabolic hormones, and aberrant insulin and glucagon signaling in mouse brain.^27^, ^29^

The present study tested the possibility that pharmacological treatment with the brain penetrant FRAX486,^25^, ^26^ a small synthetic molecule that was reported to selectively inhibit PAKs,^25^ might rescue CDD‐related neurobehavioral alterations in a *Cdkl5*‐KO mouse model. Preliminary in vitro evaluation was performed to verify whether treatment with FRAX486 was able to rescue neuronal alterations in the complete absence of *Cdkl5*. Based on the promising results obtained, we next assessed FRAX486 therapeutic efficacy in vivo on heterozygous *Cdkl5*‐KO female (*Cdkl5*‐Het) mice, the model that better reproduces the genetic and hormonal milieu of CDD patients.^30^ The same treatment regimen that improved the neurobehavioral phenotype in a mouse model of Fragile X syndrome, a disorder of monogenic origin with several clinical features in common with CDD,^25^ was applied. Given the emerging role of PAKs in glucose homeostasis regulation, FRAX486 effects on glucose metabolism and the oxidative stress status were also addressed in the CDD mouse model.

To verify the activation status of PAKs in CDD mouse brain and FRAX486 effects thereon, phosphorylation levels at sites which are considered indices of PAKs activation [serine 144 (PAK1 or αPAK), serine 141 (PAK2 or γPAK) and serine 139 (PAK3 or βPAK)] were also assessed. Given its emerging crucial role in the proper regulation of the cytoskeleton during neurite outgrowth,^17^ we also verified whether phosphorylation of PAK1 at threonine‐212 (P‐PAK1 Thr212) was affected by CDKL5 dysfunction and tested FRAX486 treatment effects thereon.

## 2 MATERIALS AND METHODS

*Mouse colony*. Experimental subjects were derived from the *Cdkl5*‐KO strain developed by^6^ and were obtained by crossing *Cdkl5*‐Het female mice and wild‐type (wt) or hemizygous male mice. The day of birth was designated as postnatal day (P) 0 and animals with 24 h of age were considered as P1 old animals. After weaning at P25‐30, mice were housed according to sex in groups of 2–5 per cage on a 12 h light/dark cycle in a temperature‐controlled environment (21 ± 1°C and relative humidity at 60 ± 10%) with a complete pellet diet (Altromin, 1324–10 mm pellets, Germany) and tap water provided ad libitum. All experimental procedures were conducted in conformity with the European Communities Council Directive (10/63/EU) as well as the Italian law (26/2014) and approved by the Italian Ministry for Health.

*Genotyping*. DNA was prepared as previously described.^11^ The Cdkl5 alleles were identified through PCR using two sets of primers. Primer set 1 (5' primer: 5'‐ ACG‐ATA‐GAA‐ATA‐GAG‐GAT‐CAA‐CCC‐3' and 3' primer: 5'‐CCC‐AAG‐TAT‐ACC‐CCT‐TTC‐CA‐3') yields a product of 240 bp identifying the wt allele. Primer set 2 (5' primer same as for primer set 1 and 3' primer: 5'‐CTG‐TGA‐CTA‐GGG‐GCT‐AGA‐GA 3') yields a product of apparent size 344 bp identifying the null allele. PCR products were separated by electrophoresis through a 2% NuSieve 3:1 agarose gel (#50090, Lonza Bio Science, Switzerland) containing 0.1 μl/ml GelRed™ (#41003, Biotium, USA) and examined under UV light.

*Drug*. FRAX486 was supplied by DBA (#HY‐15542B, DBA, Italy) and stored at −20°C, protected from light. FRAX486 was dissolved in either 20% (weight/volume) hydroxypropyl‐β‐cyclodextrin (#H107, Sigma‐Aldrich, USA) in saline or DMSO (ctrl; #D2650, Sigma‐Aldrich, USA) for in vivo and in vitro experiments, respectively.

### 2.1 In vitro treatment

#### Primary hippocampal cultures and drug treatment

Primary hippocampal neurons were prepared from P1 old wt and *Cdkl5*‐KO mice (*N* = 3–4 per group), as previously described.^31^ On day 7, in vitro (DIV7) differentiated hippocampal cultures were treated with 100 nM of FRAX486, a dose exceeding PAKs IC_50_,^25^ or ctrl and fixed 48 h later (Figure S1a).

#### Immunofluorescence

Immunostaining was performed using rabbit polyclonal anti‐MAP2 (1:100; #AB5622, Merck Millipore, USA) and mouse monoclonal anti‐PSD95 (1:200; #Ab2723 Abcam, UK) antibodies. Detection was performed using rabbit FITC‐conjugated (1:200; #111–095‐045, Jackson ImmunoResearch Laboratories, Inc., USA) and mouse Cy3‐conjugated (1:200; #115–165‐062, Jackson ImmunoResearch Laboratories, Inc., USA) secondary antibodies. Fluorescent images were acquired using a Nikon Eclipse TE600 microscope equipped with a Nikon Digital Camera DXM1200 ATI System (Nikon Instruments, Inc., USA). Neurite outgrowth was analyzed using the image analysis system Image‐Pro Plus as previously described.^31^ The degree of synaptic innervation was evaluated by counting the number of PSD95+ puncta on proximal dendrites and expressed as the number of PSD95+ puncta per 20 μm of length. A total of 30–40 neurons for each condition was evaluated.

#### Image analysis and quantification

Acquired images of MAP2 staining, specifically identifying dendrites, were analyzed thanks to the “Sholl Analysis” plugin of the FiJi software (htpp://Fiji.sc).^32^ Neurons were outlined to exclude adjacent cells or areas of nonspecific immunoreactivity. For each cell, densitometric thresholds were set to remove background labelling and identify detailed cellular processes. Values of starting radius (10 μm) to exclude neuronal soma and radius step size (10 μm) were used. The number of processes intersecting each ring was provided by the software for each cell and total number of intersections was obtained. A mean total number of intersections indicative of the branching complexity was calculated using *N* = 3–4 samples and 8–10 neurons per sample.

### 2.2 In vivo treatment

#### 2.2.1 Experiment 1

##### 2.2.1.1 Experimental design and drug treatment

*Cdkl5*‐Het mice were treated once daily for 5 consecutive days via subcutaneous (s.c.) injections of either 20 mg/Kg of FRAX486 or ctrl (*N* = 12 per group) starting at 8:00 am, as previously described.^25^ Wt mice were treated with ctrl solution only (*N* = 15). Mice were tested at an advanced stage of the disease (14–16‐month‐old^33^) in order to assess whether FRAX486 exerted beneficial effects on overt symptomatology. Of note, systemic injections of the drug at the chosen dose have been previously demonstrated to provide stable FRAX486 brain levels exceeding PAKs IC_50_ as early as 1h after its administration throughout 24 h later. A battery of tests was performed to assess FRAX486 effects on behavioral alterations in CDD mice (Figure S1b) 6 h after the s.c. injection, when this drug is near to reach its peak in the brain tissue.^25^ Similarly, on the 5^th^ day of the treatment, 6 h from the last s.c. injection, mice were sacrificed and whole blood was collected from the trunk into heparinized tubes to evaluate reactive oxidizing species (ROS) production and hippocampi were dissected and immediately frozen for subsequent analyses.

##### 2.2.1.2 Estrous cycle monitoring

To control for estrous cycle of *Cdkl5*‐Het and wt mice at the time of hippocampus collection (Figure S1b), a vaginal smear was collected as previously described.^34^ The relative ratio of cell types observed in smears stained with methylene blue was used to distinguish the four phases of rodent estrous cycle, as previously described.^35^

##### 2.2.1.3 Behavioral analyses

Behavioral testing took place 6 h after the s.c. injection, during the dark phase of the light/dark cycle (Figure S1b) and was carried out by experimenters blind to the mouse genotype and treatment. Mice were experimentally naïve.

###### Contextual fear conditioning (FC) test

The FC test was carried out to evaluate FRAX486 effects on *Cdkl5‐*Het mice learning and memory functions, as previously described.^36^ The task consisted of both a training and a testing phase, performed on the 3^rd^ and 4^th^ days of treatment respectively, 6 h after the daily s.c. injection (Figure S1b). During training, mice were left undisturbed for 180 s (baseline, BL) in the FC apparatus (a 25 × 30 × 30 cm Plexiglas box with an electrified grid floor, placed inside a sound‐proof cubicle, Coulbourn Instruments, USA) and then exposed for three times to a 30‐s‐long acoustic stimulus (CS; 2000 Hz, 90 dB) associated during the last 2 s with a single footshock (US; 0.7 mA). A 60 s inter‐trial interval (ITI) was used between the three CS + US pairings. Fear memory for the conditioning context was evaluated 24 h later. Each mouse was re‐exposed to the FC apparatus for 180 s, during which no CS or US were delivered (Test). The apparatus was cleaned with 70% ethanol among animals, and the brightness was kept at about 10 lux. Both training and testing phases were video‐recorded and subsequently scored by means of an automated software (ANY‐maze software Version 6.3, Stoelting, USA). Contextual fear memory was assessed by measuring the time spent freezing (freezing score on threshold = 30; off threshold = 40; minimum freeze duration = 1 s) during the BL and the test.

###### Open field test

To evaluate the effects of treatment on locomotor activity in a novel environment and anxiety‐like behavior, the open field test was performed on the 5^th^ day of treatment, 3 h after the last s.c. injection as previously described (Figure S1b).^37^ Mice were individually placed in the testing apparatus, a squared arena (40 × 40 cm) enclosed by black walls (35 cm high), with a white floor, at a brightness of 13 lux. The session started with the animal being placed in one corner of the arena and lasted 15 min. The floor of the apparatus was cleaned with 70% ethanol after each testing session. Total distance traveled in the whole arena and total time spent in the central area (20 × 20 cm) were measured with a tracking software (ANY‐maze software Version 6.3, Stoelting, USA) as indicators of locomotor activity and anxiety‐like behavior, respectively.

###### Dowel test

To evaluate motor coordination, mice were subjected to the dowel test as in Ref. [38] on the 5^th^ day of treatment, after the open field test (Figure S1b). The metallic round dowel used (9 mm in diameter and 35 cm long) was mounted horizontally 50 cm above a 5–cm deep sawdust bedding. At the beginning of the test, each mouse was placed in the middle of the dowel so that the length of its body was parallel to it. Latency to fall from the dowel was recorded, with a cut‐off of 30 s. Each mouse repeated the test twice, with an ITI of at least 10 min. If mice were able to walk along the dowel and get off it, or to stay on it without falling for 30 s, they received the maximum score. The average of the latency to fall from the dowel in the two trials was considered a measure of motor coordination and balance.

###### General health (GH) assessment

The GH of the experimental mice was qualitatively evaluated by a trained observer blind to mouse genotype and treatment on the last day of treatment (Figure S1b), at the end of behavioral testing, as previously described,^39^, ^40^ with little modification. Briefly, mice received a score (ranging from 0 = normal appearance to 4 = highly compromised) for each of the following symptoms: gait, mobility, breathing, kyphosis, fur, hindlimb clasping, tremors, seizures occurrence, and general condition. The individual scores for each category were subsequently averaged to obtain a semiquantitative measure of symptom status or GH score. The body weight of the animals was also measured.

##### 2.2.1.4 Measurement of ROS levels in whole blood

Trunk blood was collected into heparinized tubes on the 5^th^ day of the treatment (Figure S1b), 6 h from the last s.c. injection, to evaluate ROS levels (*N* = 6–13 per group). The oxidation of the spin probe 1‐hydroxy‐3‐carboxypyrrolidine (CPH, dissolved in degassed phosphate buffer, pH 7.4, and extensively treated with Chelex‐100 to avoid metal contamination) to the correspondent 3‐carboxy‐proxyl radical (CP^•^)^41^ was monitored by electron paramagnetic resonance (EPR). The formation of CP^•^ is not specific to a singular oxidant but is suitable for screening the totality of ROS (among which O2^•^, ^•^OH, peroxynitrite, transition metal‐catalyzed reactions) produced in biological samples. If the intensity of CP^•^ is significantly increased, the presence of a pro‐oxidant status is suggested. Briefly, CPH (0.5 mM, #ALX‐430‐078, ENZOlife Sciences, Italy) was added to 100 μl of whole blood of wt or *Cdkl5*‐Het mice and the intensity of CP^•^ was measured after 20 min at 37°C. Samples were drawn up into a gas‐permeable Teflon tube with 0.81 mm internal diameter and 0.05 mm wall thickness (Zeus Industrial Products, Inc., USA). The Teflon tube was folded four times, inserted into a quartz tube, and fixed to the cavity (4108 TMH) of a Bruker ECS 106 EPR spectrometer equipped with a variable temperature unit (ER4111VT). Spectrometer conditions were: modulation frequency, 100 kHz; microwave frequency, 9.4 GHz; microwave power, 20 mW; gain, 1 × 104; modulation amplitude, 1G; conversion time, 20.5 ms; time constant, 82 ms; sweep time, 21 s; and number of scans, 1.

##### 2.2.1.5 Western blotting (WB)

WB analysis was conducted on hippocampi collected 6 h from the last s.c. injection (*N* = 4–11 per group). The hippocampal formation was homogenized in ice‐cold RIPA buffer supplemented with 1 mM PMSF (#93482, Sigma‐Aldrich, USA), and with 1% protease (#P8340, Sigma‐Aldrich, USA) and phosphatase inhibitor cocktail (#P0044, Sigma‐Aldrich, USA). Protein concentration was determined using the Lowry method.^42^ Protein extracts were immediately processed in WB or kept frozen (−80°C) until assayed. Equivalent amounts of protein (50 μg) were subjected to electrophoresis on a BOLT Bis‐Tris Plus gel (#NW04127BOX, Thermo Fisher Scientific, USA) and transferred to a Hybond ECL nitrocellulose membrane (#10600008, Amersham ‐ GE Healthcare Life Sciences). The following primary antibodies were used: rabbit polyclonal anti‐phospho‐PAK1/2/3 (phospho‐Ser144/141/139) (1:500; #A27148PI AceBiolab, Taiwan), rabbit polyclonal anti‐phosho‐PAK1 (Ser204) (1:1000; #E‐AB‐21232, Elabscience, USA), rabbit polyclonal anti‐phosho‐PAK1 (Thr212) (1:1000; #E‐AB‐21179, Elabscience, USA), rabbit polyclonal anti‐PAK1 (1:1000; #E‐AB‐32498, Elabscience, USA), rabbit polyclonal anti‐PSD95 (1:1000; #Ab18258, Abcam, UK), rabbit polyclonal anti‐synaptophysin 1 (1:1000; #101002, SY SY, Germany), rabbit polyclonal anti‐GAPDH (1:5000; #G9545, Sigma‐Aldrich, USA), and mouse monoclonal anti‐β‐Actin (1:5000; #A5441, Sigma‐Aldrich, USA). A HRP‐conjugated goat anti‐rabbit IgG (1:5000; #111–035‐003, Jackson ImmunoResearch Laboratories, Inc., USA) secondary antibody was used. Membranes were probed with the antibody for the phosphorylated form of the analyzed protein, stripped with the Restore™ Stripping Buffer (#21063, Thermo Fisher Scientific, USA) following the manufacturer's instructions, and then re‐probed with the antibody for the nonphosphorylated forms of the same protein. Densitometric analysis of digitized images was performed using Chemidoc XRS Imaging Systems and Image Lab™ Software (Bio‐Rad, USA).

#### 2.2.2 Experiment 2

##### 2.2.2.1 Experimental design and drug treatment

An independent cohort of symptomatic *Cdkl5*‐Het mice and wt littermates (*N* = 3–4 per group) that had not undergone behavioral assessment was treated once daily for 5 consecutive days via s.c. injections of either 20 mg/Kg of FRAX486 or ctrl, to evaluate FRAX486 treatment effects on spine density/maturation (Figure S1c). Since dendritic spine abnormalities are fully manifested at an early stage of the disease,^43^ mice were treated at 6–8 months of age. Animals were sacrificed 6 h after the last injection and brains were quickly removed, cut along the midline and Golgi‐stained using the FD Rapid Golgi Stain TM Kit (#PK401, FD NeuroTechnologies, USA) as previously described.^30^, ^43^

##### 2.2.2.2 Histological analysis

Hemispheres were immersed in the impregnation solution containing mercuric chloride, potassium dichromate, and potassium chromate and stored at room temperature in darkness for 2–3 weeks. Hemispheres were cut with a microtome in 100 μm thick coronal sections that were directly mounted onto gelatin‐coated slides and were air‐dried at room temperature in the dark for an additional 2–3 days. After drying, sections were rinsed with distilled water and subsequently stained in the developing solution of the kit. Images were acquired using a Leica light microscope (Leica Microsystems, Germany; 100× oil immersion objective, NA 1.4). Dendritic spine density was measured by manually counting the number of dendritic spines on apical dendrites of CA1‐pyramidal neurons. In each mouse, 10–15 dendritic segments (segment length, 10–30 μm) were analyzed and the linear spine density was calculated by dividing the total number of counted spines by the length of the sampled segment. Based on their morphology, dendritic spines can be divided into five different classes, which also reflect their state of maturation (immature spines: filopodium‐like, thin‐shaped, and stubby‐shaped; mature spines: mushroom‐ and cup‐shaped). The total number of spines was expressed per 10 μm, and the number of spines belonging to each class was counted and expressed as a percentage. All steps of sectioning, imaging, and data analysis were conducted blindly.

#### 2.2.3 Experiment 3

##### 2.2.3.1 Glucose metabolism assessment

To test whether *Cdkl5*‐Het mice show abnormal blood glucose levels (glycemia) at a fully symptomatic stage, an independent cohort of symptomatic *Cdkl5*‐Het and wt littermates (9–12‐month‐old; *N* = 4–5 per group^33^) were tested in the glucose tolerance test (GTT) and the insulin sensitivity test (IST). Glycemia was measured in the blood collected through tail vein incision using a commercial glucometer (Wellion Luna, Med Trust, Italy).

###### GTT

After a 15‐h overnight fasting (from 7:30 pm to 10:30 am) mice were intra‐peritoneally (i.p.) loaded with 2 g/kg body weight of d‐glucose (#G8644, 10% d‐glucose solution; Sigma‐Aldrich, USA). Glycemia was measured at 0 (baseline), 30, 60, 120, and 180 min after i.p. injection.^44^

###### IST

One week later, mice were starved for 5 h (from 9:00 am to 2:00 pm), and glycemia was measured immediately before (0, baseline) and 15 min after an i.p. injection with 0.4 U/kg body weight of human recombinant insulin (#025707035, 100 U/ml solution, Humulin, Eli‐Lilly, Italy).^44^

##### 2.2.3.2 FRAX486 treatment effects on glucose metabolism

A separate cohort of symptomatic *Cdkl5*‐Het and wt mice (9–12‐month‐old; *N* = 7–10 per group) was tested to confirm the hypoglycemic profile of *Cdkl5*‐Het mice in fasting conditions and to verify whether FRAX486 treatment may rescue the impaired glucose metabolism in CDD (Figure S1d). In the first week, *Cdkl5*‐Het and wt mice were treated for 5 consecutive days via s.c. injections of ctrl solution. Glycemia was measured through tail vein incision under both basal conditions and after 5 h of fasting (from 9:00 am to 2:00 pm) on the 5^th^ day of treatment. The following week, *Cdkl5*‐Het mice received once daily, for 5 consecutive days, s.c. injections of 20 mg/kg of FRAX486, while wt mice received ctrl solution, starting at 8:00 am. Glycemia was measured as in the first week.

### 2.3 Cell line overexpressing CDKL5

#### 2.3.1 Maintenance and plasmid transfection

293 T cell line was maintained in Dulbecco Modified Eagle Medium (#21969–035, DMEM, Gibco BRL, USA) supplemented with 10% heat‐inactivated FBS, 2 mM of glutamine, and antibiotics (penicillin, 100 U/ml; streptomycin, 100 μg/ml), in a humidified atmosphere of 5% of CO_2_ at 37°C. Cell medium was replaced every 3 days and the cells were sub‐cultured once they reached 90% confluence. Cells were transfected with pCMV14‐3xFLAG‐hCDKL5 carrying the CDKL5_1 isoform that generates a protein of 960 amino acids (107 kDa), pCMV14‐3xFLAG‐hCDKL5 carrying the CDKL5_1 kinase dead isoform K42R and pCMV14‐3xFLAG empty backbone as a control (#E4901, Sigma‐Aldrich, USA) using Metafectene Easy Plus (T090, Biontex, Germany).

#### 2.3.2 WB

Twenty‐four hours after transfection cells were processed for WB (*N* = 3 per group). Total proteins from 293 T cells transfected with wt CDKL5 or empty vector were lysed in ice‐cold RIPA buffer (50 mM Tris–HCl, pH 7.4, 150 mM NaCl, 1% Triton‐X100, 0.5% sodium deoxycholate, 0.1% SDS) supplemented with 1 mM PMSF (#93482, Sigma‐Aldrich, USA), and with 1% protease (#P8340, Sigma‐Aldrich, USA) and phosphatase inhibitor cocktail (#P0044, Sigma‐Aldrich, USA). Protein concentration was determined using the Bradford method.^45^ The following primary antibodies were used: rabbit polyclonal anti‐phosho‐PAK1 (Ser204) (1:1000; #E‐AB‐21232, Elabscience, USA), rabbit polyclonal anti‐phosho‐PAK1 (Thr212) (1:1000; #E‐AB‐21179, Elabscience, USA), rabbit polyclonal anti‐PAK1 (1:1000; #E‐AB‐32498, Elabscience, USA). A HRP‐conjugated goat anti‐rabbit IgG (1:5000; #111–035‐003, Jackson ImmunoResearch Laboratories, Inc., USA) secondary antibody was used. For PAK1 WBs, membranes were probed with the antibody for the phosphorylated form of the analyzed protein, stripped with the Restore™ Stripping Buffer (#21063, Thermo Fisher Scientific, USA) following the manufacturer's instructions, and then re‐probed with the antibody for the nonphosphorylated forms of the same protein.

### 2.4 Statistical analyses

Statistical analysis was performed with IBM SPSS Statistics Version 26.0. Normality and homoscedasticity were evaluated using Shapiro–Wilk's and Levene's tests, respectively. Datasets with normal distribution were analyzed for significance using Student's *t*‐test or one‐way analysis of variance (ANOVA), with Dunnett's test for post hoc comparisons, setting *Cdkl5*‐KO, ctrl or *Cdkl5*‐Het, ctrl as control group. Repeated measures ANOVA (setting the experimental group as between factor and repeated measurements as within factor) followed by Tukey's post hoc test was also used. Animals identified as outliers by the use of Grubb's test were excluded from the analyses.^46^ Datasets without normal distribution were analyzed for significance with Kruskal–Wallis test and Dunn's test for post hoc comparisons. A probability level of *p* < 0.05 was considered to be statistically significant. Results are presented as mean ± standard error of the mean (SEM, bar plots) or as 10^th^, 25^th^, 50^th^ (median), 75^th^, and 90^th^ percentiles (box plots). Statistical analyses results are reported in Tables S1 and S2.

## 3 RESULTS

### 3.1 FRAX486 treatment restores dendritic development and density of PSD95+ puncta in *Cdkl5*‐KO neurons

**FIGURE 1:**
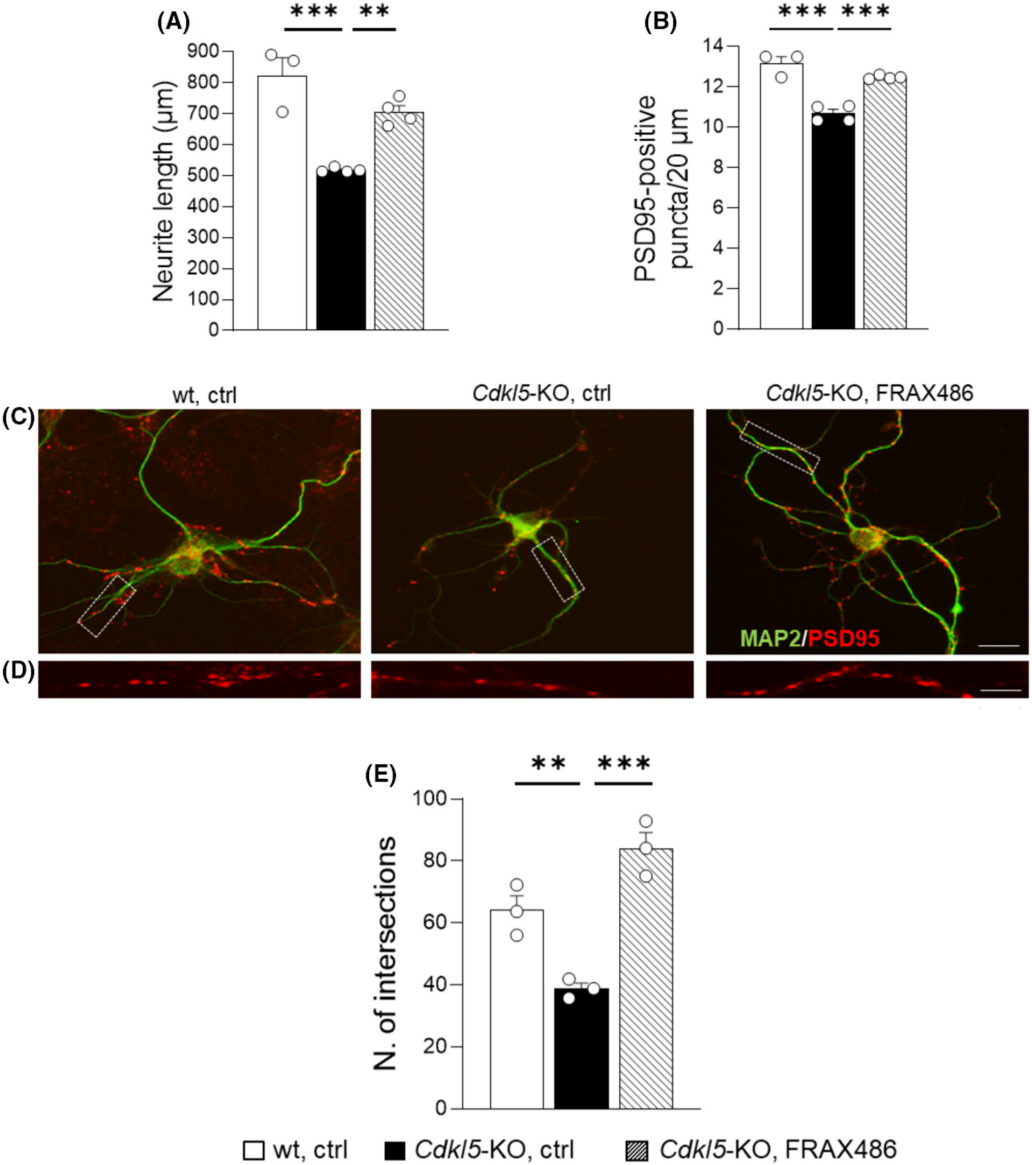
Treatment with FRAX486 normalizes neurite outgrowth in an in vitro model of CDD. Primary hippocampal neurons were prepared from 1‐day‐old (P1) wild‐type (wt) and *Cdkl5* knockout (*Cdkl5‐*KO) mice. On the 7th day, in vitro (DIV7) differentiated hippocampal cultures were treated with 100 nM FRAX486 or control solution (ctrl) and fixed 48 h later (DIV9) for immunofluorescence analysis. (A) Quantification of neurite outgrowth of microtubule‐associated protein 2 (MAP2)‐positive (1:100; Merck Millipore) in hippocampal neurons was done using the image analysis system Image‐Pro Plus. FRAX486 treatment rescued the abnormal dendritic development in *Cdkl5*‐KO neurons. (B) The degree of synaptic innervation was evaluated by counting the number of PSD95+ puncta (1:200; Abcam) on proximal dendrites and expressed as the number of PSD95+ puncta per 20 μm of length. FRAX486 treatment rescued the reduced density of PSD95+ puncta in *Cdkl5*‐KO neurons. (C and D) Representative fluorescence images of differentiated MAP2‐positive hippocampal neurons (C; scale bar: 10 μm) and proximal dendrites immunopositive for PSD95 (D; scale bar: 1 μm) of wt and *Cdkl5‐*KO mice. (E) Quantification of dendritic branching represented as the total number of intersections. FRAX486 normalized the number of intersections in *Cdkl5*‐KO neurons. Statistical significance was assessed using one‐way ANOVA (main effect of experimental group) followed by Dunnett's post hoc test (setting *Cdkl5*‐KO, ctrl as control group). Data are mean ± SEM. **p < 0.01, ***p < 0.001; *N* = 3–4 per group

To assess whether FRAX486 treatment rescues CDKL5‐dependent neurodevelopmental alterations, we first tested its effects on primary hippocampal cultures from *Cdkl5*‐KO mice, a reliable in vitro disease‐related model that recapitulates many of the most striking neuronal CDD phenotypes.^30^, ^31^, ^32^, ^43^ In fact, in line with previous reports, hippocampal neurons from *Cdkl5*‐KO mice showed reduced neurite outgrowth (*p* < 0.001 for *Cdkl5*‐KO, ctrl vs. wt, ctrl; Figure 1A,C), reduced number of PSD95+ puncta (*p* < 0.001 for *Cdkl5*‐KO, ctrl vs. wt, ctrl; Figure 1B, D), and reduced neuronal branching (*p* < 0.01 for *Cdkl5*‐KO, ctrl vs. wt, ctrl; Figure 1E) compared with wt controls. Treatment with FRAX486 improved all these parameters in *Cdkl5*‐KO neurons (*p* < 0.01, *p* < 0.001, and *p* < 0.001 for *Cdkl5*‐KO, ctrl vs. *Cdkl5*‐KO, and FRAX486, respectively; Figure 1).

### 3.2 FRAX486 treatment rescues behavioral alterations in *Cdkl5*‐Het mice at an advanced stage of the disease

Based on the promising results of the in vitro experiments, we daily treated with FRAX486 for 5 days *Cdkl5*‐Het mice, a female mouse model of CDD that recapitulates the genetic and hormonal milieu of patients, thus representing the most valid model for the disorder.^11^, ^30^ A battery of behavioral tests was carried out to evaluate treatment efficacy (Figure S1b).

**FIGURE 2:**
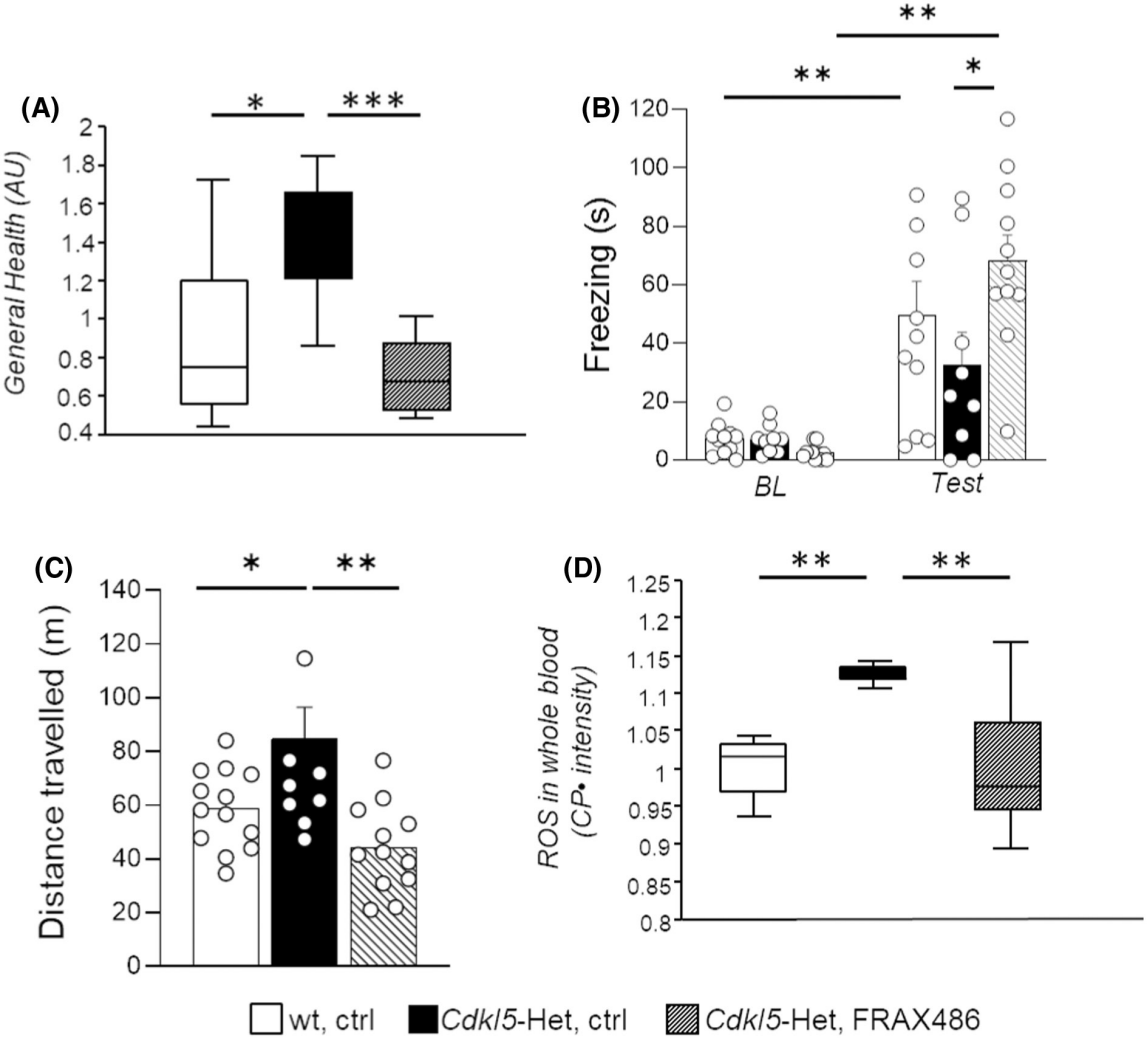
FRAX486 treatment improves behavioral abnormalities, general health status and oxidative stress levels in the blood of *Cdkl5*‐Het female mice at an advanced stage of the disease, a validated CDD mouse model. *Cdkl5* heterozygous (*Cdkl5‐*Het) female mice received subcutaneous (s.c) injections with FRAX486 (20 mg/kg, daily) or control (ctrl) solution for 5 days. Wild‐type (wt) littermates were treated only with ctrl. A battery of tests was performed to assess drug effects on behavior and whole blood was collected to measure the levels of reactive oxidizing species (ROS). (A) The general health status was qualitatively evaluated by a trained observer blind to mouse genotype and treatment. Briefly, mice received a score (ranging from 0 = normal appearance to 4 = highly compromised) for each of the following symptoms: gait, mobility, breathing, kyphosis, fur, hindlimb clasping, tremors, seizures occurence and general condition. The individual scores for each category were subsequently averaged to obtain a semiquantitative measure of symptom status, called general health score (measured in average units, AU). FRAX486 treatment normalized the general health status of *Cdkl5*‐Het female mice. (B) Time spent freezing during the baseline (BL) and the testing phase (Test) of the fear conditioning task was automatically monitored (Any Maze v.6.3, Stoelting). FRAX486 treatment improved the fear memory of *Cdkl5*‐Het female mice. (C) Distance traveled during a 15‐min‐long open field task was measured by the means of a tracking software (Any Maze v.6.3, Stoelting) and considered as a measure of locomotor activity in a novel environment. FRAX486 treatment rescued the hyperactive profile of *Cdkl5*‐Het female mice. (D) Nitroxyl 3‐carboxy‐proxyl radical (CP^•^) intensity was measured in whole blood by electron paramagnetic resonance (EPR) and considered as an index of ROS production; data are normalized to wt, ctrl values. FRAX486 treatment rescued the pro‐oxidant status that occurs in the blood of *Cdkl5*‐Het mice. Statistical significance was assessed using one‐way ANOVA (main effect of experimental group) followed by Dunnett's post hoc test (setting *Cdkl5*‐Het, ctrl as control group). Repeated measures ANOVA (setting experimental group as between factor and repeated measurements as within factor) followed by Tukey's post hoc test was also performed. In case of deviation from normality and homoschedasticity, data were analyzed by means of nonparametric Kruskal–Wallis test followed by Dunn's post hoc test. Bar plots represent mean ± SEM (B, C) while boxplots represent the 10th, 25th, 50th (median), 75th, and 90th percentiles (A, D). *p < 0.05, **p < 0.01, ***p < 0.001; *N* = 12–15 per group (A–C) or *N* = 6–13 per group (D)

*Cdkl5*‐Het mice at an advanced stage of the disease showed the presence of phenotypic abnormalities, confirming severely impaired general health conditions (*p* < 0.05 for *Cdkl5*‐Het, ctrl vs. wt, ctrl; Figure 2A). The impaired health status of *Cdkl5*‐Het mice was completely rescued by a 5‐day long treatment with FRAX486 (*p* < 0.001 for *Cdkl5*‐Het, ctrl vs. *Cdkl5*‐Het, FRAX486; Figure 2A). No differences were found in the body weight of the experimental subjects (Table S2) and the evaluation of the estrous cycle confirmed that the mice were in *diestrus* and in *metestrus* in a balanced way within each of the three experimental groups.

*Cdkl5*‐Het female mice also displayed fear memory deficits in the contextual fear conditioning test. Indeed, while wt controls significantly increased the time spent freezing after conditioning (*p* < 0.01 for Test vs. BL; Figure 2B), *Cdkl5*‐Het females did not freeze significantly more during context re‐exposure compared with baseline. Systemic treatment with FRAX486 was able to increase the time *Cdkl5*‐Het females spent freezing during the testing phase, thus improving their memory deficits (*p* < 0.01 for *Cdkl5*‐Het, FRAX486 Test vs. BL and *p* < 0.05 for *Cdkl5*‐Het, ctrl vs. *Cdkl5*‐Het, FRAX486 during the Test; Figure 2B).

*Cdkl5*‐Het mice also showed a hyperactive profile when exposed to a new environment, as demonstrated by the longer distance traveled compared with wt controls during the open field task (*p* < 0.05 for *Cdkl5*‐Het, ctrl vs. wt, ctrl; Figure 2C). Interestingly, treatment with FRAX486 normalized the altered locomotor profile of CDD mice, restoring wt‐like levels (*p* < 0.01 for *Cdkl5*‐Het, ctrl vs. *Cdkl5*‐Het, FRAX486; Figure 2C). The analysis of time spent in the center/intimidating area of the arena did not reveal any differences among groups on the anxiety‐like behavior in this task (Table S2).

We could not evidence any difference in motor coordination or balance among groups in the dowel test, with all mice showing comparable latencies to fall (Table S2).

### 3.3 FRAX486 treatment counteracts the increased oxidative stress status displayed by *Cdkl5*‐Het mice

Given that mitochondrial dysfunction and oxidative stress also occur in CDD mouse models,^9^, ^10^, ^11^ we evaluated ROS production in the blood by EPR^41^ at the end of the 5 days of treatment (Figure S1b). We confirmed that a pro‐oxidant status occurs in the blood of *Cdkl5*‐Het mice, as demonstrated by the increased rate of CP^•^ formation compared with wt littermates (*p* < 0.01 for *Cdkl5*‐Het, ctrl vs. wt, ctrl; Figure 2D). After 5 days of FRAX486 administration, ROS levels in whole blood of *Cdkl5*‐Het were decreased and normalized to the level of wt controls (*p* < 0.01 for *Cdkl5*‐Het, ctrl vs. *Cdkl5*‐Het, FRAX486; Figure 2D).

### 3.4 *Cdkl5*‐Het mice show fasting‐induced hypoglycemia, that is normalized after treatment with FRAX486

**FIGURE 3:**
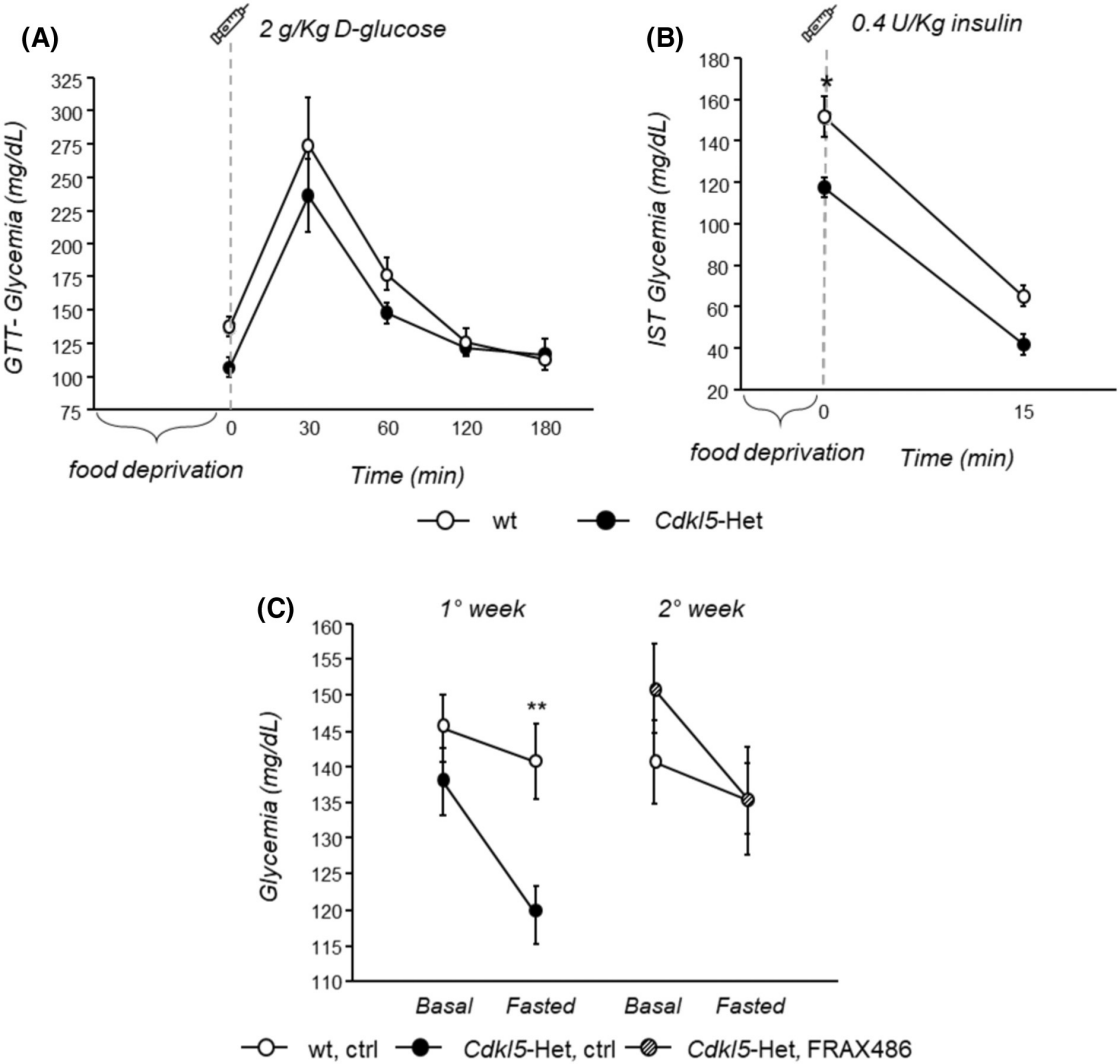
Treatment with FRAX486 rescues the aberrant blood glucose homeostasis displayed by symptomatic *Cdkl5*‐Het mice. (A) Glucose tolerance test (GTT). Blood glucose levels (glycemia) in *Cdkl5* heterozygous (*Cdkl5*‐Het) female mice and wild‐type (wt) littermates were measured using a commercial glucometer (Menarini diagnostic) from tail vein blood collected at 0 (baseline), 30, 60, 120 and 180 min following intraperitoneal (i.p.) injection with 2 g/kg body weight d‐glucose (10% D glucose solution; Sigma Aldrich) after overnight fasting. No significant genotype differences were found in the GTT. (B) Insulin sensitivity test (IST). One week later, mice were i.p. injected with human recombinant insulin solution (0.4 U/kg body weight, Humulin, Eli‐Lilly) after being food‐deprived for 5 h. Blood was collected from tail vein at 0 (baseline) and 15 min after insulin administration and glycemia was measured as in (a). *Cdkl5‐*Het mice showed fasting‐induced hypoglycemia. (C) *Cdkl5*‐Het and wt mice from a separate cohort were treated for 5 consecutive days via s.c. injections of ctrl and, on the 5^th^ day of treatment, glycemia was measured through tail vein incision under both basal conditions and after 5 h of fasting (1° week). The following week (2° week), *Cdkl5*‐Het mice received once daily, for 5 consecutive days, s.c. injections of 20 mg/kg of FRAX486, while wt mice received ctrl solution, and glycemia was measured as in the first week. A 5‐day‐long treatment with FRAX486 completely rescued the fasting‐induced hypoglycemia of *Cdkl5*‐Het mice, restoring wt‐like glycemic levels. Statistical significance was assessed using repeated measures ANOVA (setting genotype as between factor and repeated measurements as within factor) followed by Tukey's post hoc test. Data are mean ± SEM. *p < 0.05, **p < 0.01; *N* = 4–5 per group (A, B) or *N* = 7–10 per group (C)

To characterize the glucose metabolism of *Cdkl5*‐Het mice at an advanced stage of the disease, the GTT and IST were performed. In the GTT, we found that *Cdkl5*‐Het and wt controls showed similar response profiles, with blood glycemic levels reaching a peak 30 min after glucose administration in both genotypes (Figure 3A). Interestingly, *Cdkl5*‐Het mice showed reduced glycemia compared with wt littermates in the IST (*p* = 0.002 for *Cdkl5*‐Het vs. wt; Figure 3B). This genotype difference was particularly marked at baseline, on food‐deprived animals (*p* < 0.05 for *Cdkl5*‐Het vs. wt at min 0; Figure 3B). To confirm the fasting‐induced hypoglycemic profile of fully symptomatic *Cdkl5*‐Het mice, glycemia was measured after a 5 h‐fasting in a separate cohort of mice. We confirmed that *Cdkl5*‐Het mice show a hypoglycemic profile when fasted compared with wt controls [Mean glycemia (mg/dL) ± SEM: basal: wt = 145.400 ± 4.681; *Cdkl5*‐Het = 137.857 ± 4.611; fasted: wt = 140.800 ± 5.183; *Cdkl5*‐Het = 119.286 ± 4.069; data not shown]. These findings provide innovative evidence that aberrant CDKL5 functioning may disrupt mechanisms that regulate glucose homeostasis. To test whether alterations in PAKs activation may be involved in the impaired glucose metabolism of *Cdkl5*‐Het mice, we next assessed whether a 5‐day long treatment with FRAX486 may restore the abnormal blood glucose levels in fasted *Cdkl5*‐Het mice. We found that a 5‐day long treatment with FRAX486 completely rescued the fasting‐induced hypoglycemia of *Cdkl5*‐Het mice (*p* < 0.01 vs. wt, ctrl; Figure 3C), restoring wt‐like glycemic levels (Figure 3C).

### 3.5 FRAX486 treatment restores spine maturation and synapse development in *Cdkl5*‐Het mouse brain

**FIGURE 4:**
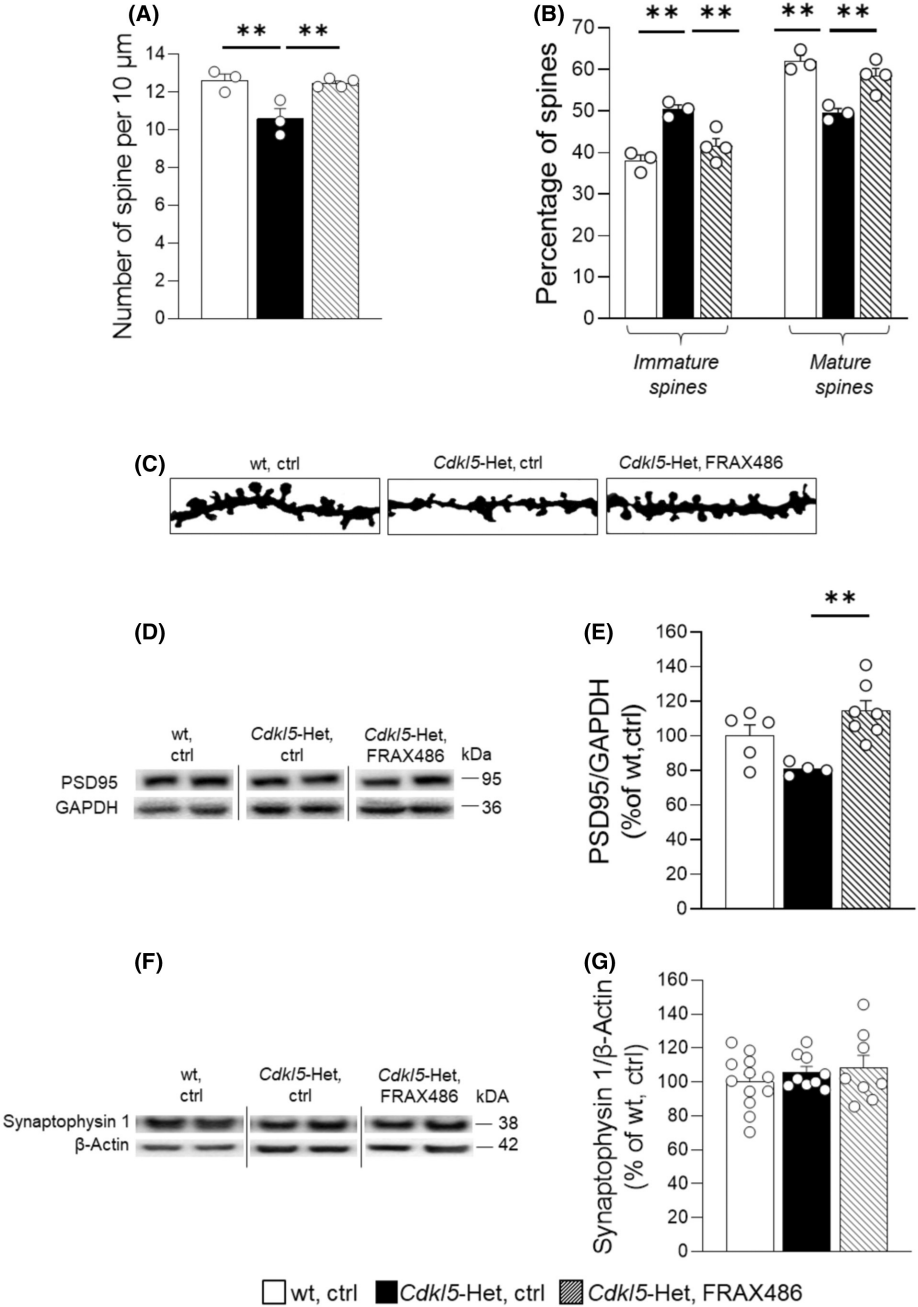
Treatment with FRAX486 rescues spine maturation and PSD95 expression in the hippocampus of *Cdkl5*‐Het mice. Symptomatic *Cdkl5* heterozygous (*Cdkl5*‐Het) female mice received daily subcutaneous (s.c.) injections of FRAX486 (20 mg/kg, daily) or control (ctrl) solution for 5 consecutive days. Wild‐type (wt) littermates received only ctrl. Six hours after the last injection hippocampal tissue was dissected for histological analyses. (A) Dendritic spine density was measured on Golgi‐stained brain coronal sections by manually counting the number of dendritic spines on apical dendrites of CA1‐pyramidal neurons. In each mouse, 10–15 dendritic segments (segment length, 10–30 μm) were analyzed and the linear spine density was calculated by dividing the total number of counted spines by the length of the sampled segment. FRAX486 treatment increased the defective spine density in *Cdkl5*‐Het female mice. (B) Based on their morphology, dendritic spines can be divided into 5 different classes (immature spines: Filopodium‐like, thin‐shaped, and stubby‐shaped; mature spines: Mushroom‐ and cup‐shaped), which also reflect their state of maturation. The number of spines belonging to each class was counted and expressed as a percentage in relation to the total number of protrusions in neurons. FRAX486 treatment rescued spine imbalance in CA1 pyramidal neurons of *Cdkl5*‐Het mice. (C) Examples of Golgi‐stained dendritic branches of CA1 pyramidal neurons of one animal from each experimental group. Scale bar: 1 μm. (D, E) PSD95 levels were normalized to total GAPDH contents and expressed as a percentage of those of wt, ctrl mice. The following primary antibodies were used: Rabbit polyclonal anti‐PSD95 (1:1000; Abcam), rabbit polyclonal anti‐GAPDH (1:5000; Sigma‐Aldrich). Densitometric analysis of digitized images was performed using Chemidoc XRS Imaging Systems and Image Lab™ Software (Bio‐Rad). FRAX486 treatment increased the reduced PSD95 levels in hippocampal extracts of *Cdkl5*‐Het mice. Immunoblots were assembled to show examples from two animals of each experimental group. (F, G) Synaptophysin 1 levels were normalized to total β‐Actin contents and expressed as a percentage of those of wt, ctrl mice. The following primary antibodies were used: Rabbit polyclonal anti‐synaptophysin 1 (1:1000, SYSY) and mouse monoclonal anti‐β‐actin (1:5000, Sigma‐Adrich). Densitometric analysis of digitized images was performed using Chemidoc XRS Imaging Systems and Image Lab™ Software (Bio‐Rad). No genotype or treatment effects were found in synaptophysin 1 levels in hippocampal extracts of experimental mice. Immunoblots were assembled to show examples from two animals of each experimental group. Statistical significance was assessed using one‐way ANOVA (main effect of experimental group) followed by Dunnett's post hoc test (setting *Cdkl5*‐Het, ctrl as control group). Data are mean ± SEM. **p < 0.01; *N* = 3–4 per group (A, B), *N* = 4–7 per group (E), *N* = 8–12 per group (G)

To test whether a systemic treatment with a FRAX486 rescues CDD‐related dendritic spine abnormalities in vivo,^47^ spine density and morphology of dendritic spines were evaluated in Golgi‐stained CA1 pyramidal neurons. In agreement with previous data,^43^ *Cdkl5*‐Het mice showed a reduction in spine density and in the percentage of mature spines and a greater proportion of immature spines (*p* < 0.01 for *Cdkl5*‐Het, ctrl vs. wt, ctrl; Figure 4A–C) compared with their wt counterparts. In line with the in vitro results, treatment with FRAX486 significantly increased spine density and rescued the imbalance in dendritic spine shape between immature and mature ones in CA1 pyramidal neurons of Cdkl5‐Het mice (*p* < 0.01 for *Cdkl5*‐Het, ctrl vs. *Cdkl5*‐Het, FRAX486; Figure 4A–C).

To evaluate whether FRAX486 treatment has positive effects on synaptic innervation, we performed WB analysis to examine PSD95 and synaptophysin 1 levels in hippocampal extracts of wt and *Cdkl5*‐Het mice. *Cdkl5*‐Het mice showed reduced PSD95 levels compared with their wt littermates, although the difference missed statistical significance. Treatment with FRAX486 significantly increased PSD95 levels in hippocampal extracts of *Cdkl5*‐Het mice (*p* < 0.01 for *Cdkl5*‐Het, ctrl vs. *Cdkl5*‐Het, FRAX486; Figure 4D,E), thus restoring wt‐like levels. No effects of genotype or treatment were found on synaptophysin 1 hippocampal levels (Figure 4F,G).

### 3.6 FRAX486 treatment restores the abnormal PAKs phosphorylation in *Cdkl5*‐Het mouse hippocampus

PAKs are finely regulated through phosphorylation at multiple sites. To shed some light on the mechanism of action of FRAX486 in CDD mouse brain, we analyzed the levels of phosphorylation of three autophosphorylation sites which mark the active state conformation of PAKs (phospho‐serine 144/141/139, P‐PAKs)^48^ and of the threonine 212 of PAK1 (P‐PAK1 Thr212), an important site for the regulation of cytoskeleton dynamics in neurons.^17^

**FIGURE 5:**
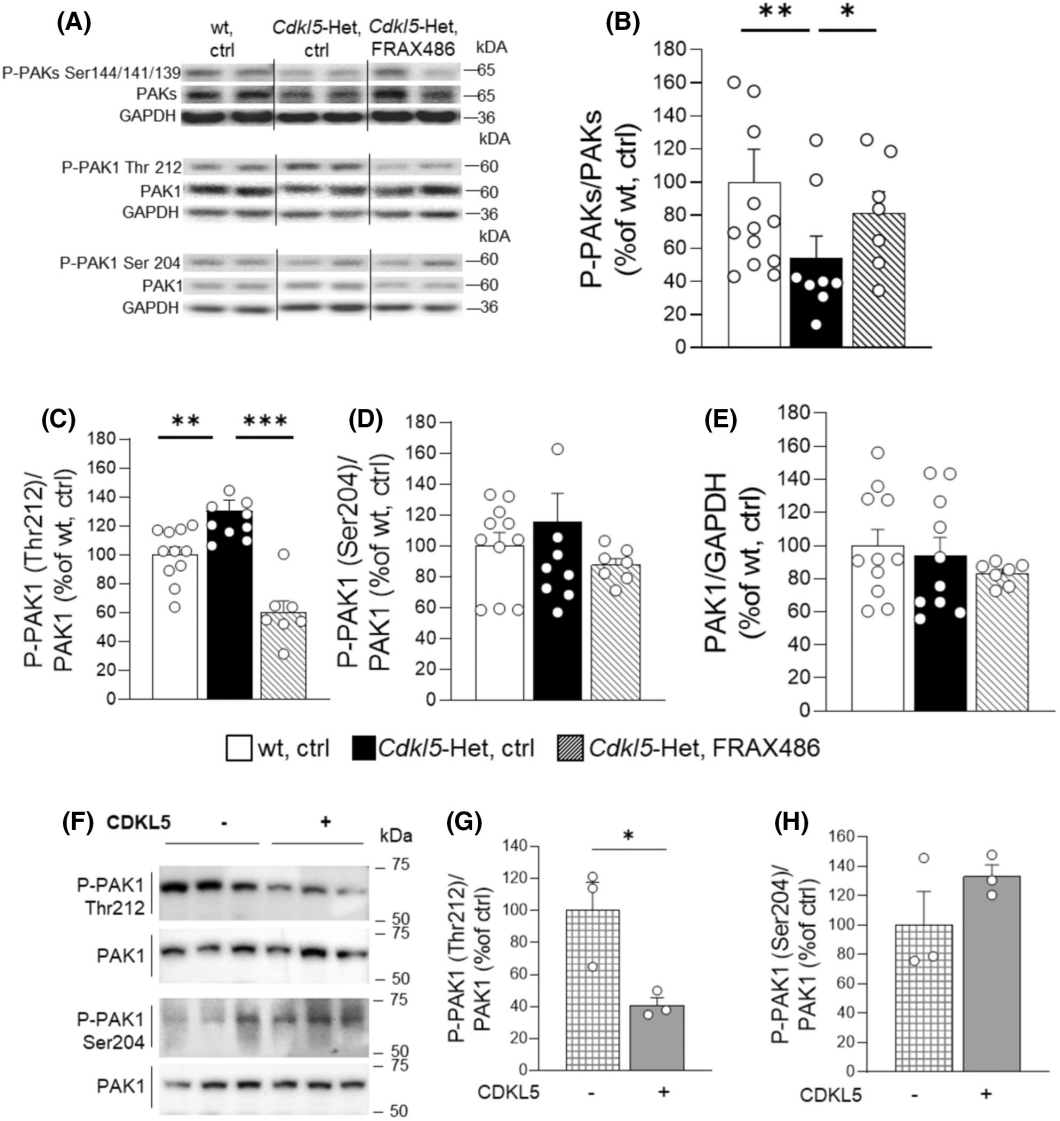
FRAX486 treatment rescues the abnormal phosphorylation levels of PAKs in *Cdkl5*‐Het mouse hippocampus. Fully symptomatic *Cdkl5* heterozygous (*Cdkl5*‐Het) female mice received daily subcutaneous (s.c.) injections of FRAX486 (20 mg/kg, daily) or control (ctrl) for 5 consecutive days. Wild‐type (wt) littermates received only ctrl solution. Six hours after the last injection hippocampal tissue was dissected for western blot analyses of phospho‐PAK1/2/3 (P‐PAKs) normalized to total PAK1/2/3 content (PAKs), phospho‐PAK1 threonine‐212 (P‐PAK1 Thr212), and phospho‐PAK1 serine‐204 (P‐PAK1 Ser204) levels in hippocampal homogenates. P‐PAK1 levels were normalized to total PAK1 contents, while total PAK1 levels were normalized to GAPDH levels in hippocampal extracts. (A) Immunoblots were assembled to show examples from two animals of each experimental group. (B) FRAX486 normalized the reduced P‐PAKs/PAKs ratio in hippocampal extracts from *Cdkl5*‐Het mice at wt‐like levels. (C) FRAX486 rescued the increased levels of P‐PAK1 at Thr212 in hippocampal extracts from *Cdkl5*‐Het mice. (D and E) No significant difference was found in the analysis of P‐PAK1 at Ser204 or in total PAK1 contents. (F–H) To substantiate the suggested link between disrupted *Cdkl5* functionality and aberrant phosphorylation of phospho‐PAK1 on threonine‐212 (P‐PAK1Thr‐212), 293 T cells were transfected with pCMV14‐3xFLAG‐hCDKL5 carrying the CDKL5_1 isoform that generates a protein of 960 amino acids (107 kDa) and pCMV14‐3xFLAG empty backbone as a control (ctrl, Sigma Aldrich) using Metafectene Easy Plus (Biontex). Twenty‐four hours later, transfection cells were processed for western blotting. (F) Immunoblots from technical replicates of P‐PAK1 (Thr212, Ser204) levels in 293 T total protein extracts from control and CDKL5‐transfected cells. (G) Western blot analysis revealed a reduction of P‐PAK1 at Thr212 in the presence of the overexpression of CDKL5. (H) No significant difference was found in the analysis of phospho‐PAK1 serine‐204 (P‐PAK1 at Ser 204). P‐PAK1 levels were normalized to total protein contents in cells. Data in B–E are expressed as a percentage of the values of wt, ctrl while in G,H as a percentage of those of cells transfected with the empty vector (ctrl). Statistical significance was assessed using one‐way ANOVA (main effect of experimental group) followed by Dunnett's post hoc test (setting *Cdkl5*‐Het, ctrl as control group) for B–E and with Student's *t*‐test for G,H. Data are mean ± SEM. *p < 0.05, **p < 0.01, ***p < 0.001; *N* = 7–11 per group (B–E) or *N* = 3 (G, H)

We found that the levels of P‐PAKs/PAKs ratio, which provides an index of the net functionality of the kinase, was shifted toward decreased phosphorylation for *Cdkl5*‐Het mouse hippocampus compared with wt controls (*p* < 0.01 for *Cdkl5*‐Het, ctrl vs. wt, ctrl; Figure 5A,B). Surprisingly, the administration of FRAX486, which is considered a potent PAKs inhibitor,^25^ significantly increased P‐PAKs/PAKs ratio in *Cdkl5*‐Het hippocampal homogenates, thus restoring wt‐like levels (*p* < 0.05 for *Cdkl5*‐Het, ctrl vs. *Cdkl5*‐Het, FRAX486; Figure 5A,B).

WB analyses on hippocampal extracts from *Cdkl5*‐Het mice also showed increased levels of P‐PAK1 Thr212 in comparison with wt mice (*p* < 0.01 for *Cdkl5*‐Het, ctrl vs. wt, ctrl; Figure 5A,C). Interestingly, FRAX486 treatment significantly reduced P‐PAK1 Thr212 levels in *Cdkl5*‐Het mice (*p* < 0.001 for *Cdkl5*‐Het, ctrl vs. *Cdkl5*‐Het, FRAX486; Figure 5A,C). The levels of PAK1 autophosphorylation at a site that is not directly related to PAK1 kinase activity (P‐PAK1 Ser204) were also assessed as a control.^49^ No effects of genotype or treatment were observed on hippocampal P‐PAK1 Ser204 levels or in total PAK1 protein levels (Figure 5A,D,E).

Given the fundamental role played by this phosphorylation site in the regulation of cytoskeleton dynamics^17^ and the suggested involvement of this process in CDD pathogenesis,^7^ we also addressed the existence of a potential relationship between Cdkl5 functionality and P‐PAK1 Thr212 in Cdkl5 overexpressing cells. Indeed, we found that cells transfected with a Cdkl5‐containing vector depicted decreased phosphorylation of PAK1 at Thr212 site (*p* < 0.05 relative to control; Figure 5F). No significant differences were detected at serine 204 (Figure 5F,H) or in total PAK1 protein levels (Table S2). As expected, 293 T cells transfected with a kinase‐dead CDKL5 protein (K42R) did not differ in phosphorylation of PAK1 at the Thr212 site from controls, confirming that wild type CDKL5, but not the mutated form, affects PAK1 Thr212 phosphorylation (Figure S2).

## 4 DISCUSSION

The present study demonstrates that systemic treatment with FRAX486 exerts compelling beneficial effects on neurobehavioral alterations in a female mouse model of CDD and rescues the increased levels of ROS and fasting‐induced hypoglycemia in CDD mouse blood. Interestingly, these effects were accompanied by a normalization of PAKs hypoactivation in CDD mouse hippocampus. The first evidence that CDKL5 dysfunction affects the phosphorylation of Thr‐212 PAK1, a site that has emerged as crucial for the proper regulation of neuronal morphology,^17^ is also provided.

The evidence demonstrating that CDKL5 plays a crucial role in brain development and function is overwhelming. However, the mechanisms leading to the neurological symptoms associated with CDD have not been completely clarified.^5^ We here provide innovative evidence that treatment with FRAX486 rescues the reduced dendritic branching and the synaptic defects in a CDD mouse model, thus normalizing the altered neuronal phenotype. Of note, these beneficial effects were observed in a simplified in vitro model as well as in *Cdkl5*‐Het female mouse brain, the model which more closely resembles the genetic and hormonal milieu of CDD patients.^11^, ^30^ Furthermore, the rescue was obtained in differentiated neurons, when CDD‐related neuronal defects are already well‐established, as well as in mice at an advanced stage of the disease, suggesting a beneficial effect of the treatment on the defective neural maintenance that occurs in CDD.^50^ Interestingly, treatment with FRAX486 specifically normalizes PSD95, without affecting synaptophysin 1, which is not altered in CDD mouse brain, suggesting that this molecule provides a selective effect at the defective post‐synaptic level. Taken together, these results suggest a crucial role of PAKs‐dependent pathways in CDD neuropathology and support their relevance as novel therapeutic targets for this disorder.

Surprisingly, however, we found that systemic treatment with FRAX486 normalized PAKs hypoactivation in CDD mouse hippocampus. Given the reported role of the molecule as a PAKs inhibitor,^25^ the FRAX486‐induced PAKs activation in CDD mouse brain was an unexpected finding. It must however be noted that such a surprising effect was exerted under dysfunctional baseline conditions, i.e., in CDD mouse hippocampus, where a PAKs hypoactivation was observed. It is therefore conceivable that the FRAX486 treatment might have compensated for some alterations deriving from the lack of CDKL5 rather than directly increase PAKs activation. Yet, given that the mechanisms of action of this molecule have not been clarified so far, it is difficult to hypothesize which kind of compensatory mechanisms may have been activated by the systemic treatment. It is also worth noting that the PAKs hypoactivity we found in the hippocampus of CDD female mice was contrary to our expectations. In fact, PAKs hyperactivation was previously reported in *Cdkl5*‐KO male mice in the same brain area,^11^ suggesting that PAKs activation status may be sex‐dependently affected in the absence of CDKL5. We cannot however exclude that the difference from previous study could be due to the antibodies, with the one used in the former study appearing as selective for PAK2^11^ rather than for PAK1/2/3. Further studies are certainly needed to clarify these important points.

While the mechanisms underlying PAKs hypoactivation in CDD mouse brain and FRAX486 effects thereon have to be further explored, it is reliable to speculate that the beneficial effects of the treatment with FRAX486 on CDD‐related dysfunction in mouse neurons may be attributed to a rescue of altered PAKs‐dependent cytoskeleton dynamics. PAKs are in fact critical regulators of cytoskeleton dynamics underlying neuronal morphological changes and plasticity and, in particular, the Thr‐212 phosphorylation site of PAK1 has been proposed to affect the cross‐talk between microtubules and microfilaments during the forward movement and turning of neuronal growth cones,^17^ a process that was shown to be deranged in CDD neurons.^51^ Given the emerging role of microtubule alterations in CDD pathogenesis,^7^ further studies will have to verify the impact of PAKs pharmacological modulation on microtubule stability defects in CDD mouse neurons. Interestingly, CDKL5 deficiency and overexpression exerted opposite effects on Thr‐212 PAK1 phosphorylation, suggesting a direct link between CDKL5 and PAK1‐dependent modulation of cytoskeleton dynamics. As the mechanisms underlying neuronal defects in CDD have not been clarified yet, present data provide an interesting molecular candidate to be further explored.

Importantly, the normalization of the altered neuronal phenotype was accompanied by the rescue of CDD‐related behavioral alterations in the CDD mouse model, suggesting the potential therapeutic value of this treatment strategy for CDD. In particular, FRAX486 treatment restored fear memory defects in CDD female mice, confirming a number of evidence suggesting a tight link between PAKs alterations and cognitive dysfunction.^52^, ^53^ FRAX486 treatment also rescued the general health status and the hyperactive profile in CDD female mice at an advanced stage of the disease. Since a recent study has demonstrated that inhibiting the dopamine transporter with methylphenidate rescues the hyperactivity in a CDD mouse model,^54^ it may be interesting in future studies to assess FRAX486 treatment effects on the defective dopaminergic system in CDD mouse brain. Previous investigations have in fact suggested the involvement of PAK1 in dopamine neuron loss and Parkinson's disease.^55^

Another major finding of the present study regards the demonstration that CDD female mice at an advanced stage of disease display alterations in blood glucose levels that are rescued by systemic treatment with FRAX486. In fact, despite recent studies have suggested an important role for CDKL5 in the regulation of energy metabolism,^9^, ^11^ this is, to the best of our knowledge, the first study addressing the presence of alterations in peripheral glucose homeostasis in a CDD mouse model. Moreover, data on the glucose metabolism in CDD patients are inconsistent and no systematic evaluation has been performed to verify whether an impairment occurs.^56^, ^57^ We focused our attention on this specific metabolic profile since PAKs‐dependent modulation of cytoskeleton remodeling has been highlighted as an important regulator of glucose homeostasis.^27^ Furthermore, severe hypoglycemia is frequently associated with seizures, one of the most impairing symptoms in CDD patients,^1^, ^58^ thus stressing the need for a better understanding of its potential relevance for CDD pathogenesis and seizure control.

We also provide evidence that the aberrant pro‐oxidant status occurring in CDD mouse blood can be substantially improved by systemic treatment with FRAX486. This result is consistent with previous studies demonstrating an increase in oxidative stress markers in CDD patients‐derived fibroblasts and plasma.^9^, ^10^ No study had however addressed so far oxidative stress status in *Cdkl5*‐Het mice. Interestingly, numerous evidence have highlighted a tight link between metabolic dysfunction and the increased generation of ROS,^59^ with studies suggesting a causal relationship between oxidative stress levels and aberrant insulin signaling/sensitivity. Particularly relevant to our research is the observation that antioxidant treatments can normalize fasting‐induced hypoglycemia and increase insulin sensitivity.^60^ It will be interesting in future studies to verify whether enhanced oxidative stress‐dependent insulin signaling may occur in CDD mice, that can be rescued by PAKs normalization or antioxidant treatments. Although this hypothesis is intriguing, at the moment we cannot exclude that independent mechanisms account for the reported rescue of the alterations in pro‐oxidant status and glucose homeostasis.

In conclusion, the present study identifies PAKs as promising therapeutic targets for CDD, a highly debilitating disorder for which no cure exists. Although the surprising effect of the treatment with FRAX486 on PAKs activation status in CDD mouse brain challenges the translational value of this molecule, present data provide innovative evidence that PAKs activating drugs may represent promising therapeutic candidates for CDD. The emerging role of PAKs as crucial regulators of glucose homeostasis is also confirmed, further stressing the need for PAKs expression levels to be finely regulated both in the brain and in the periphery. Present results also shed some new light on CDD metabolic alterations, by demonstrating that glucose homeostasis is compromised in a female mouse model of the disorder. Furthermore, increased levels of reactive oxidizing species in whole blood of CDD mice were first highlighted, that could be exploited as markers of treatment efficacy.

## AUTHOR CONTRIBUTIONS

CF, MCT, and BDF designed experiments. MCT and LC performed behavioral analyses. CF and GM performed in vitro studies and western blots. NM performed in vitro image analysis and quantification. MCQ and DP performed EPR analysis. LC and CU performed data analysis. DP, EC, AF, and BDF provided resources and supervised data collection and analysis. BDF, LC, and CU wrote the article. BDF conceived the study, acquired funding, and administered the project. All the authors reviewed the article.

## CONFLICT OF INTEREST

The authors declare no conflict of interest.

## Supporting information


Appendix S1

Appendix S2

Appendix S3

## Data Availability

The data of this study are available from the corresponding author upon reasonable request.
