# Supplementary figures and images for: Treatment with FRAX486 rescues neurobehavioral and metabolic alterations in a female mouse model of CDKL5 deficiency disorder
Source: CNS Neurosci Ther. 2022 Aug 6;28(11):1718–32. doi: 10.1111/cns.13907 (PMC9532911; doi:10.1111/cns.13907)

Fig. S2

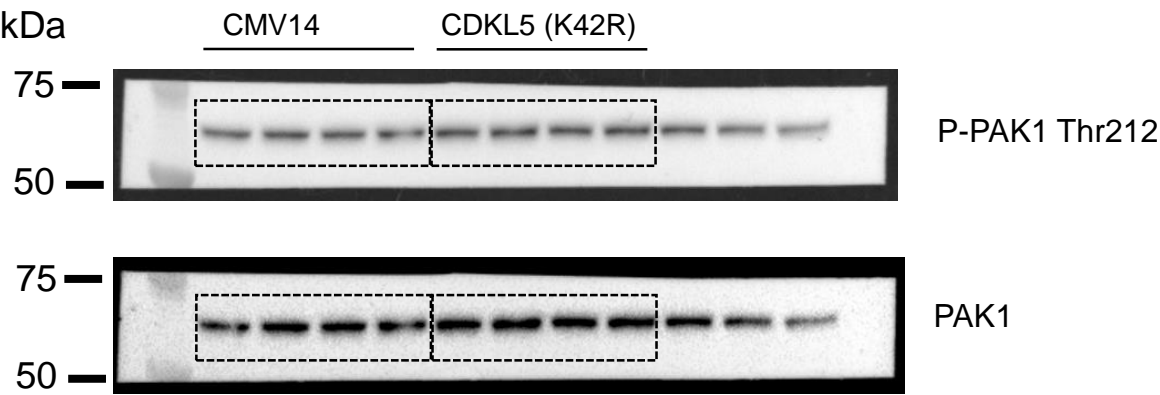

Supplement: Supplementary file 1 — Appendix S1 [file CNS-28-1718-s003.pdf]
