# Supplementary material for: Treatment with FRAX486 rescues neurobehavioral and metabolic alterations in a female mouse model of CDKL5 deficiency disorder
Source: CNS Neurosci Ther. 2022 Aug 6;28(11):1718–32. doi: 10.1111/cns.13907 (PMC9532911; doi:10.1111/cns.13907)
Supplement: Supplementary file 2 — Appendix S2 [file CNS-28-1718-s001.docx]

**Supplement to:**

**Treatment with FRAX486 rescues neurobehavioural and metabolic alterations in a female mouse model of CDKL5 Deficiency Disorder**

**Supplementary Table S1: summary of statistical results**

|  | Variable | | Statistical analysis | *Post hoc* comparisons |
| --- | --- | --- | --- | --- |
| *Neuronal phenotype* | Neuritic lenght  (Fig. 1a) | | One-way ANOVA  Experimental group: F_2,8_=25.85; p<0.001 | Dunnett’s test  *Cdkl5*-KO, ctrl:  - vs wt, ctrl: p<0.001  - vs *Cdkl5*-KO, FRAX486: p<0.01 |
|  | PSD95+ puncta  (Fig. 1b) | | One-way ANOVA  Experimental group: F_2,8_=40.04; p<0.001 | Dunnett’s test  *Cdkl5*-KO, ctrl:  - vs wt, ctrl: p<0.001  - vs *Cdkl5*-KO, FRAX486: p<0.001 |
|  | Number of intersections  (Fig. 1e) | | One-way ANOVA  Experimental group: F_2,6_=29.915; p=0.001 | Dunnett’s test  *Cdkl5*-KO, ctrl:  - vs wt, ctrl: p<0.01  - vs *Cdkl5*-KO, FRAX486: p<0.001 |
| *Behavioural assessment* | GH score  (Fig. 2a) | | Kruskal–Wallis test  Experimental group: H_2_=15.36; p<0.001 | Dunn’s test  *Cdkl5*-Het, ctrl:  - vs wt, ctrl: p<0.05  - vs *Cdkl5*-Het, FRAX486: p<0.001 |
|  | Freezing in FC task  (Fig. 2b) | | Repeated Measures ANOVA  Phase*Experimental group interaction: F_2,28_=3.37; p=0.049 | Tukey’s test  wt, ctrl (BL) vs wt, ctrl (Test): p<0.01; *Cdkl5*-Het, FRAX486 (BL) vs *Cdkl5*-Het, FRAX486 (Test): p<0.01; *Cdkl5*-Het, ctrl (Test) vs *Cdkl5*-Het, FRAX486 (Test): p<0.05 |
|  | Distance travelled in  OF task  (Fig. 2c) | | One-way ANOVA  Experimental group: F_2,32_=8.00; p=0.002 | Dunnett’s test  *Cdkl5*-Het, ctrl:  - vs wt, ctrl: p<0.05  - vs *Cdkl5*-Het, FRAX486: p<0.01 |
| *Oxidative stress status assessment* | ROS levels  (Fig. 2d) | | Kruskal–Wallis test  Experimental group: H_2_=10.84; p=0.004 | Dunn’s test  *Cdkl5*-Het, ctrl:  - vs wt, ctrl: p<0.01  - vs *Cdkl5*-Het, FRAX486: p<0.01 |
| *Glucose homeostasis* | Glycemia in GTT test  (Fig. 3a) | | Repeated Measures ANOVA  Glycemia*Genotype interaction: F_1,32_=0.89; p=0.480 | / |
|  | Glycemia in IST test  (Fig. 3b) | | Repeated Measures ANOVA  Genotype: F_1,7_=23.73; p=0.002  Glycemia: F_1,7_=141.95; p<0.001  Glycemia*Genotype interaction: F_1,7_=0.57; p=0.476 | Tukey’s test  wt (0 min) vs *Cdkl5*-Het (0 min): p<0.05 |
|  | FRAX486 effects  on glycemia  (Fig. 3c) | | Repeated Measures ANOVA (1°week) Genotype*Phase: F_1,15_=2.86; p=0.110  Repeated Measures ANOVA (2°week) Genotype*Phase: F_1,15_=0.43; p=0.53 | Tukey’s test  wt, ctrl (Fast) vs *Cdkl5*-Het, ctrl (Fast): p<0.01 |
| *Dendritic spine maturation* | Number of spines  (Fig. 4a) | | One-way ANOVA  Experimental group: F_2,7_=11.23; p=0.007 | Dunnett’s test  *Cdkl5*-Het, ctrl:  - vs wt, ctrl: p<0.01  - vs *Cdkl5*-Het, FRAX486: p<0.01 |
|  | Percentage of spines  (Fig. 4b) | Immature spines | One-way ANOVA  Experimental group: F_2,7_=15.42; p=0.003 | Dunnett’s test  *Cdkl5*-Het, ctrl:  - vs wt, ctrl: p<0.01  - vs *Cdkl5*-Het, FRAX486: p<0.01 |
|  |  | Mature spines | One-way ANOVA  Experimental group: F_2,7_=15.42; p=0.003 | Dunnett’s test  *Cdkl5*-Het, ctrl:  - vs wt, ctrl: p<0.01  - vs *Cdkl5*-Het, FRAX486: p<0.01 |
|  | PSD95/GAPDH  (Fig. 4e) | | One-way ANOVA  Experimental group: F_2,13_=7.79; p=0.006 | Dunnett’s test  *Cdkl5*-Het, ctrl vs *Cdkl5*-Het, FRAX486: p<0.01 |
|  | Synaptophysin 1/β-Actin  (Fig. 4g) | | One-way ANOVA  Experimental group: F_2,26_=0.744; p=0.485 | / |
| *Phosphorylation of PAK1* | P-PAKs/PAKs in hippocampal homogenates (Fig. 5b) | | One-way ANOVA  Experimental group: f_2,25_=5.828;  p=0.008 | Dunnett’s test:  *Cdkl5*-Het, ctrl:  - vs wt, ctrl: p<0.01  - vs *Cdkl5*-Het, FRAX486:  p<0.05 |
|  | P-PAK1 (Thr212)/PAK1 in hippocampal homogenates  (Fig. 5c) | | One-way ANOVA  Experimental group: f_2,25_=22.61;  p=0.001 | Dunnett’s test:  *Cdkl5*-Het, ctrl:  - vs wt, ctrl: p<0.01  - vs *Cdkl5*-Het, FRAX486:  p<0.001 |
|  | P-PAK1 (Ser204)/PAK1 in hippocampal homogenates  (Fig. 5d) | | One-way ANOVA  Experimental group: F_2,25_=1.01; p=0.382 | Dunnett’s test  *Cdkl5*-Het, ctrl vs *Cdkl5*-Het, FRAX486: NS |
|  | PAK1/GAPDH in hippocampal homogenates  (Fig. 5e) | | One-way ANOVA  Experimental group: F_2,25_=0.72; p=0.499 | / |
| *CDKL5 overexpression* | P-PAK1 (Thr212)/PAK1 in cells transfected  (Fig. 5g) | | Student's *t*-test  Genotype*: t*_4_=3.24; p=0.032 | / |
|  | P-PAK1 (Ser204)/PAK1 in cells transfected  (Fig. 5h) | | Student's *t*-test  Genotype: *t*_4_=-1.36; p=0.245 | / |
| *Kinase-dead CDKL5 protein* | P-PAK1 (Thr212)/PAK1 in cells transfected  (Fig. S2) | | Student's *t*-test  Genotype*: t*_5_=-0.324; p=0.759 | / |

Abbreviations: wt - wild-type mice; *Cdkl5*-KO - *Cdkl5* knockout mice; *Cdkl5*-Het *- Cdkl5* heterozygous mice; ctrl - control solution; GH - general health; FC - fear conditioning; BL – baseline phase in FC task; Test – test phase in FC task; OF - open field; ROS - reactive oxygen species; GTT - glucose tolerance test; IST - insulin sensitivity test; Fast – fasted; / - not applicable.

**Supplementary Table S2: summary of non-significant results**

|  | Variable | Statistical analysis | Mean ± SD |
| --- | --- | --- | --- |
| *Behavioural assessment* | Body weight | One-way ANOVA  Experimental group: F_2,36_=1.38; p=0.266 | wt, ctrl: 25.147±1.646 g  *Cdkl5*-Het, ctrl: 25.517±2.953 g  *Cdkl5*-Het, FRAX486: 26.467±1.455 g |
|  | Time in center in OF task | One-way ANOVA  Experimental group: F_2,36_=0.62; p=0.940 | wt, ctrl: 30.848 ±30.861 %  *Cdkl5*-Het, ctrl: 32.410±32.475 %  *Cdkl5*-Het, FRAX486: 34.706±19.043 % |
|  | Fall latency in  DT task | Kruskal–Wallis test  Experimental group: H_2_=0.90; p=0.637 | wt, ctrl: 25.300±7.218 s  *Cdkl5*-Het, ctrl: 26.250±5.246 s  *Cdkl5*-Het, FRAX486: 25.250±7.421 s |
| *WB analyses* | PAK1 total levels in cells transfected | Student's *t*-test  Genotype: *t*_4_=-0.74; p=0.500 | Cells overexpressing CDKL5: 0.839±0.121 (relative to control)  control: 1±0.357 |

Abbreviations: SD – standard deviation; wt - wild-type mice; *Cdkl5*-Het *- Cdkl5* heterozygous mice; ctrl - control solution; OF – open field task; DT – dowel test.

**Supplementary Figures**

**
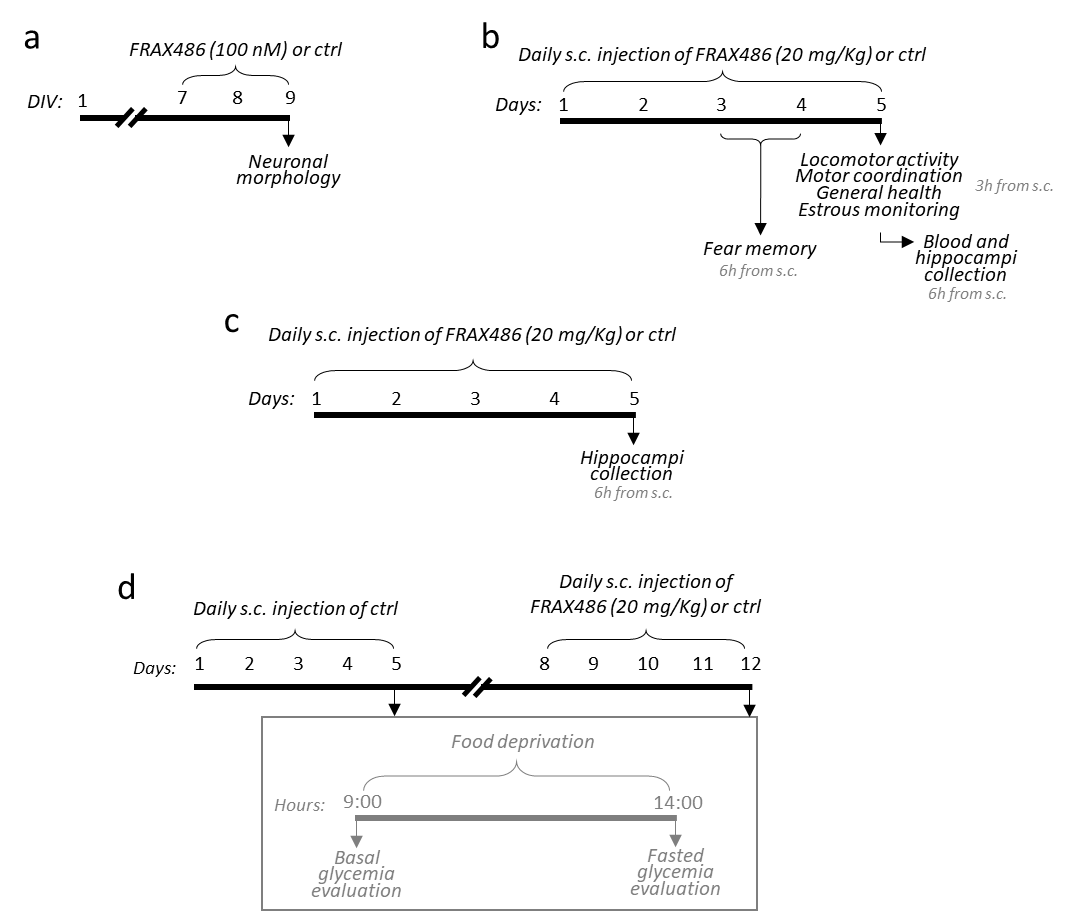
**

**Fig. S1. *In vitro* and *in vivo* FRAX486 treatment schedules. (a)** Primary hippocampal neurons were prepared from 1-day-old (P1) wild-type (wt) and *Cdkl5* knockout (*Cdkl5-*KO) mice. On the 7^th^ day *in vitro* (DIV7) differentiated hippocampal cultures were treated with 100 nM FRAX486 or control solution (ctrl) and fixed 48 h later (DIV9) for immunofluorescence analysis. **(b)** *Cdkl5* heterozygous (*Cdkl5-*Het) female mice received subcutaneous (s.c) injections with FRAX486 (20 mg/kg, daily) or ctrl for 5 days. Wt littermates were treated with ctrl solution only. A battery of tests was performed to assess drug effects on behaviour. On the 5^th^ day of treatment, 6 h after the last injection, whole blood was collected to measure the levels of reactive oxidizing species and hippocampi were dissected for western blot analyses. **(c)** A cohort of 6-8 months old *Cdkl5*-Het and wt mice was treated once daily for 5 consecutive days via s.c. injections of either FRAX486 (20 mg/Kg, daily) or ctrl, to evaluate FRAX486 treatment effects on spine density/maturation. On the 5^th^ day of treatment, 6 h after the last injection, hippocampi were dissected for histological analyses. **(d)** A cohort of 9-12 months old *Cdkl5*-Het and wt mice treated for 5 consecutive days via s.c. injections of ctrl solution, and, on the 5^th^ day of ctrl treatment, glycemia was measured through tail vein incision under both basal conditions and after a 5 h of fasting. The following week, *Cdkl5*-Het mice received s.c. injections with FRAX486 (20 mg/Kg, daily) for 5 consecutive days while wt mice received ctrl solution only. Glycemia was measured as in the first week.


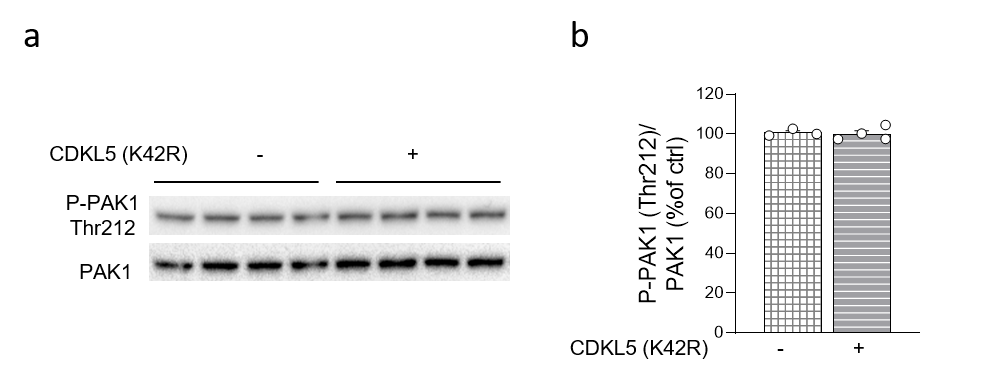


**Fig. S2. Cells transfected with a kinase dead CDKL5 protein (K42R) do not differ from controls in phosphorylation of PAK1 at Thr212 site. (a)** Immunoblots from technical replicates of P-PAK1 (Thr212) levels in 293T total protein extracts from control (-) and CDKL5-transfected cells (+). **(b)** Western blot analysis revealed no differences in P-PAK1 at Thr212. P-PAK1 levels were normalised to total protein contents in cells. Data are expressed as a percentage of the values of cells transfected with the empty vector (-). Statistical significance was assessed using Student's *t*-test. Data are mean ± SEM. N=3-4.
